# Predictors of long term use of psychiatric services of patients with recent-onset schizophrenia: 12 years follow-up

**DOI:** 10.1186/s12888-016-1186-x

**Published:** 2017-01-14

**Authors:** Víðir Sigrúnarson, Rolf W. Gråwe, Stian Lydersen, Gunnar Morken

**Affiliations:** 1 Department of Mental Health, Norwegian University of Science and Technology, Trondheim, Norway; 2 Department of Psychiatry, St. Olav`s University Hospital, Trondheim, Norway; 3 Department of Research and Development, Drug and Alcohol Treatment Health Trust in Central Norway, Trondheim, Norway; 4 Regional Centre for Child and Youth Mental Health and Child Welfare – Central Norway, Norwegian University of Science and Technology, Trondheim, Norway; 5 Department of Research and Development, Division of Mental Health, St. Olavs University Hospital, P O Box 3250, Sluppen, 7006, Trondheim, Norway

**Keywords:** Schizophrenia, Hospitalizations, Mental health service use

## Abstract

**Background:**

The aim of study was to investigate predictors of long term use of psychiatric services of patients with recent-onset schizophrenia.

**Methods:**

A cohort of 50 clinically stable patients with recent-onset schizophrenia was included in a randomized controlled trial comparing early integrated treatment with treatment as usual. Recent onset was defined as emergence of psychotic symptoms for the first time during the preceding 2 years. The follow up period was from the date of randomization and until 12 years after termination of treatment trial, 14 years forward.

**Results:**

Score on Brief psychiatric rating scale both at baseline and after 2 years of treatment, suicide attempts during 2 years of treatment and being an inpatient during 2 years of treatment were significant predictors of long term use of services.

**Conclusion:**

High score on Brief psychiatric rating scale, suicide attempts and being admitted as inpatient early in the course of schizophrenia are possible predictors of long term use of services.

**Trial registration:**

ClinicalTrials.gov NCT00184509. Registered 15 September 2005.

**Electronic supplementary material:**

The online version of this article (doi:10.1186/s12888-016-1186-x) contains supplementary material, which is available to authorized users.

## Background

Schizophrenia has been described as a chronic, relapsing disease, although with a heterogeneous course and outcome [1]. It manifests itself as a mixture of positive, negative, cognitive and mood symptoms, with general decay in function and social disability [2]. Schizophrenia is associated with increased suicide-rate [3] and reduced longevity [4]. Onset of psychotic symptoms usually occurs during adolescence or early adulthood.

The so called “critical period hypothesis” claims that in schizophrenia deterioration occurs rapidly during a period of few years following psychotic debut [5]. Subsequently the disease reaches a “plateau”, deterioration slows down or halts, and the disease reaches a level of morbidity which remains relatively stable [6]. Interventions applied at the early stages of the critical period might have the potential of preventing or diminishing the progressive deterioration and thereby improving long term prognosis [7].

Re-hospitalization following relapse is one of the costliest interventions in schizophrenia and increases the burden associated with psychotic illness [8–10]. The prediction of prognosis early in the course of illness can help developing optimal treatment strategies throughout the duration of disease course. Identifying risk factors for the use of in- and outpatient psychiatric services may have implications on clinical decisions, policy for patient care, program development, and service planning. Early identification of poor responders would allow timely adjustments to management programs and modifiable predictors may present specific treatment targets.

The aim of the study was to investigate possible baseline and 2 year predictors of long term use of psychiatric services of patients with recent-onset schizophrenia.

## Methods

### Participants and design

A cohort of 50 patients with recent-onset non-affective psychosis were included in a randomized controlled trial comparing early integrated treatment (IT) with treatment as usual (TAU). Recent onset was defined as emergence of psychotic symptoms for the first time during the preceding 2 years. TAU comprised optimal pharmacotherapy and case management, while IT also included cognitive-behavioural family treatment, which incorporated skills training, cognitive-behavioural strategies for residual psychotic and non-psychotic problems and home-based crisis management. Participants were randomly allocated using a sequence of sealed pre-numbered envelopes with group assignments according to random numbers provided by the international Optimal Treatment Project administration. Gender was stratified in blocks of varying size (between 8 and 12 and with a ratio of 3:2 of IT to TAU) ensuring that a majority of the patients received the experimental treatment. Prior to randomization written informed consent was obtained. The intervention program and the results of the trial are reported in detail elsewhere [11–14], see also the Additional file 1.

The research was carried out in compliance with the Helsinki Declaration and the study was approved by The Regional Committee for Medical and Health Research Ethics in Central-Norway.

### Recruitment

The patients were included from 1992 to 1997. The catchment area was a county in Mid-Norway with a population of about 250.000 inhabitants. At the time of inclusions, no effort was made to reduce the delay in seeking treatment in this region, but efforts were made to get referrals of all patients with recent onset psychotic disorder from psychiatric inpatient units, outpatient clinics, and general practitioners in the area. Patients who were clinically stable and were expected to reside in the county for at least 1 year after inclusion were asked to participate in the study. Patients with substance use disorders or mental retardation were excluded.

The focus of the present study was to identify predictors of long term use of services for patients with a recent-onset psychosis.

### Follow up

The follow up period was from the date of randomization until 12 years after termination of treatment trial, 14 years forward. The cohort was divided into two groups based on the number of days as psychiatric inpatients during 12 years after termination of the treatment trial. This was based on inspection of the data showing a marked dividing line in participants’ inpatient days around 100 days.

### Measures

Data on duration and number of psychiatric hospital admissions was extracted from the official Patient Administrative System, a standard system for clinical data in the health trust. Data was verified trough scrutiny and cross-checking of medical records.

Information used in this study was registered at baseline and 2 years after inclusion into the original study.

Baseline factors: Age, gender and residence. Diagnosis according to DSM-IV and overall function assessed using Global assessment of function (GAF) [15]. Psychopathology was assessed using Brief psychiatric rating scale (BPRS) [16] and Target symptoms rating scale (TSRS) [17]. Incidences and duration of hospitalisations before study entry was also recorded.

#### Factors after the 2 year treatment trial

Overall function assessed using Global assessment of function (GAF) [15] and psychopathology assessed using Brief psychiatric rating scale (BPRS) [16] and Target symptoms rating scale (TSRS) [17] at the end of the trial. Suicide attempts defined as attempts with an intention to die and adherence to psychosocial treatment and adherence to treatment with psychotropics were also registered during the 2 year trial. Incidences and duration of hospitalisations, incidences and duration of in- and outpatient coercion and contact with community or specialist health services were also registered during the 2 year treatment trial. Integrated treatment or treatment as usual according to the randomized controlled study the 2 year treatment trial. In Table 1 possible predictors of long term use of services at baseline and observations during 2 years of treatment are referred.

**Table 1:** Possible predictors of long term use of services at baseline and observations during 2 years of treatment

	Baseline	Two years
Demographics		
Age	X	
Gender	X	
Urban / Rural	X	
ASSESSMENTS		
Diagnosis	X	
Global Assessment of functioning (GAF)	X	X
Brief Psychiatric Rating Scale (BPRS)	X	X
Psychotic relapse: A 2-point increase and a score of 6 or 7 on the Target Symptoms Rating Scale (TSRS) and a score of 6 or 7 on one of the key psychotic items on the BPRS OR a 2-point increase and a score of 4 or 5 on the TSRS following a period of remission.		X
Persistent psychotic symptoms: A score of 5 or more on “hallucinations” or “unusual thoughts” on BPRS for more than 6 consecutive months		X
Attempted suicide: Attempts with intention to die		X
Use of services		
Hospitalized	Year preceding study entry	X
Days hospitalized	Year preceding study entry	X
No. of hospitalizations -		X
Involuntary admitted		X
Days involuntary admitted		X
Outpatient coercion		X
Days outpatient coercion		X
Treatment		
Integrated treatment		X
Good adherence with oral or depot antipsychotics Less than 1 month or fewer than 4 single weeks without medication		X
Good adherence with psychosocial treatment Attended at least one session per month during the treatment period		X

### Operational variables

#### During the 2 year treatment trial

“Psychotic relapse” was defined as a two-point increase and a score of six or seven on the TSRS (0–7) [17] and a score of six or seven on one of the key psychotic symptom items on the BPRS (1–7) OR a two-point increase and a score of four or five on the TSRS *following a period of remission*.

A score of five or more on “hallucinations” or “unusual thoughts” items on BPRS for more than six consecutive months was defined as having “persistent psychotic symptoms” [11]. Positive symptoms sub-score on BPRS was defined as sum score of the items “unusual thought content”, “suspiciousness”, “grandiosity”, “hallucinations”, “conceptual disorganization” and “bizarre behaviour”. Negative symptoms sub-score was defined as sum score of the items “blunted affect”, “emotional withdrawal”, “motor retardation”, “self-neglect” and “disorientation” [18]. Compliance with psychosocial treatment was considered “good” if patients attended at least one session per month during the treatment period. Patients with less than 1 month or fewer than four single weeks without medication were rated as adherent with medication. Patients receiving depot injections of antipsychotics at any time were recorded as depot users [11].

#### During the 12 year follow-up

*“Frequent users”* were defined as patients with 100 or more hospital days during the follow up period. *“Non-frequent users”* were defined as a patient with less than 100 days as psychiatric inpatients during the follow up period.

A subgroup of “frequent users”, with more than 500 inpatient days during the follow up period was defined as *“extensive users”* for further exploration.

### Missing data

Twenty four months registration on the BPRS was missing for five patients. In these cases we used last available registration. Three patients had last registration at 22 months, one at 20 months and one at 6 months.

### Analysis

#### Statistical methods

First, bivariate relationships between frequent users and the independent variables were explored. Second, possible predictors of becoming frequent users were analysed using logistic regression.

For logistic regression, general rules of thumb state that there should be at least five to ten times as many cases in the smallest group as the number of predictors [19, 20]. Hence, we included four predictors simultaneously.

#### Selection of variables

Selecting factors for inclusion in a multivariable model based on statistical significance in a bivariate analysis is not optimal as it may exclude possible confounders that impacts outcome when included in the model [21]. We therefore based our selection of variables on previous research on prognostic factors in schizophrenia and on what we considered of clinical relevance.

Age at onset and male gender are known to affect prognosis and the extent of use of services of patients with schizophrenia [22, 23], BPRS has been shown to be an applicable tool to predict length of hospital stay [24] and previous admissions have been found to predict future admissions [25]. We selected age, gender and treatment regime (Integrated treatment or treatment as usual) to be adjusted for in the logistic regression analysis of potential predictors of long term use of services.

There was a striking difference between the groups in suicide attempts during the 2 year treatment trial (zero patients attempted suicide in the non-frequent users group and five in the frequent users group) and suicidal behaviour has been found to be a predictor of psychiatric admissions [26].

#### Statistical analyses

Positive symptoms after 2 years were influenced by an extreme score in the non-frequent group and the variance of days hospitalized and number of hospitalizations during the 2 year treatment period, days involuntary hospitalized and days involuntary outpatient coercion during 2 year treatment period in the two groups, were skewed and therefore we used the non-parametric Mann-Whitney test for these variables in the bivariate analysis. Proportions were compared using the unconditional z-pooled test as recommended by Lydersen, Langaas and Bakke (2012). This test preserves the type I error and has substantially higher statistical power than Fisher's exact test in small samples.

Potential predictors for frequent users were analysed using logistic regression, with one potential predictor at a time, unadjusted, and adjusted for age, sex and treatment group (IT or TAU). Hospital days during 12 years after end of treatment were not normally distributed. As suggested by Tabatchnick & Fidell [27], we attempted the log transformation (after adding 1 to avoid trying to take the logarithm of zero) and the square root transformation. Only the square root gave acceptable approximation to the normal distribution, judged by visual inspection of QQ plots.

As secondary analyses we carried out linear regression analyses with square root transformed days in hospital during 12 years after treatment as dependent variable.

Statistical analyses were done in SPSS 20 except the unconditional z-pooled test which was done in the software http://www4.stat.ncsu.edu/~boos/exact/.

## Results

Complete 12 year follow-up data on use of services for 45 of the 50 patients were accessible from hospital records. Five patients had migrated from the region. One patient, randomized into the TAU group, died 4 years and 9 months into the follow-up period. Data from the deceased participant was included. Among the 45 patients, 21 were frequent users and 24 non-frequent users, see Table 2.

**Table 2:** Use of inpatient days through 12 years divided on Integrated treatment and Treatment as usual the two preceding years

		Integrated Treatment *N* = 28	Treatment as usual *N* = 17
Non-frequent users *N* = 21	No admittances	13	4
	0 to 100 days	3	1
Frequent users *N* = 24	100 to 500 days	6	8
	More than 500 days	6	4

Frequent users had a mean (SD) of 710 (656) days as inpatients and a median of 423 (Min 104–Max 2521), while non-frequent users had a mean (SD) of 13 (29) days and a median of zero (Min 0–Max 97), *p* < 0.001. During the 12 year follow-up period, 17 patients had no hospitalizations and 10 patients were hospitalized for more than 500 days.

### Bivariate analysis

In the bivariate analysis, gender, GAF, total score BPRS and score on BPRS positive factors at baseline were significantly associated with being a frequent user.

After the 2 years treatment trial were GAF, total score BPRS, psychotic relapse, suicide attempt, number of hospitalizations, days as inpatient, involuntary admitted, days involuntary admitted, outpatient coercion, days with outpatient coercion and not having good adherence with oral or depot antipsychotics significantly associated with being a frequent user. See Tables 3 and 4.

**Table 3:** Bivariate analysis of baseline predictors for long time use of services

	Total (*n* = 45)		Frequent users (*n* = 24)		Non-frequent users (*n* = 21)		*P*-value
Demographics							
Age. Mean (SD)	24.5 (4.5)		23.9 (4.0)		25.1 (4.9)		.36 a)
Female, No. (%)	18 (40)		13 (54)		5 (24)		.045*c)
Urban No. (%)	27 (60)		15 (63)		12 (57)		.76 c)
Diagnosis DSM-IV No. (%)							
Schizophrenia	37 (82)		20 (83)		17 (81)		-
- Schizo-affective	6 (13)		3 (13)		3 (14)		-
- Schizophreni-form	2 (5)		1 (4)		1 (5)		-
GAF baseline. Mean (SD)	49.7 (10.7)		45.8 (9.1)		54.1 (10.9)		.007*a)
BPRS baseline, 24 items. Mean (SD)	39.7 (7.6)		42.1 (7.5)		36.9 (6.9)		.020*a)
- Neg. sympt. Mean (SD)	9.4 (3.4)		9.7 (3.5)		9.1 (3.2)		.55 a)
- Pos. sympt. Median, (mean)	9 (9.6)		10.5 (10.7)		7 (8.3)		.021*b)
	Min	Max	Min	Max	Min	Max	
	6	18	6	18	6	17	
Use of services							
Hospitalized before study entry No (%)	39 (87)		20 (83)		19 (90)		.53 c)
Days hospitalized year preceding study entry. Mean (SD)	130.8 (103.2)		119.7 (98.2)		143.4 (109.7)		.45 a)

*BPRS* Brief Psychiatric Rating Scale

*GAF* Global assessment of function

*SD* Standard deviation

a) *T*-test, b) Mann-Whitney test c) Exact unconditional z-pooled test

*Statistically significant

**Table 4:** Bivariate analysis of predictors for long time services, 2 years after inclusion

	Total (*n* = 45)		Frequent users (*n* = 24)		Non-frequent users (*n* = 21)		*P*-value
Assessments							
GAF 24 months. Mean (SD)	55.2 (17.3)		49.1 (13.8)		62.1 (18.5)		.010* a)
BPRS 24 items, 2 years. Mean (SD)	36.4 (9.2)		39.8 (7.5)		32.5 (9.7)		.007* a)
-Neg. symptoms Mean (SD)	7.7 (3.7)		8.0 (3.7)		7.4 (3.7)		.63 a)
-Pos. symptoms Median (mean)	8 (9.8)		9 (10.6)		6 (8.8)		.009* b)
	Min	Max	Min	Max	Min	Max	
	6	29	6	18	6	29	
Psychotic relapse 2 years. No. (%)	23 (51)		17 (71)		6 (29)		.008* c)
Persistent psychotic sxs 0–24 months. No. (%)	11 (24)		8 (33)		3 (14)		.14 c)
Attempted suicide 2 years. No (%)	5 (11)		5 (21)		0 (0)		.032* c)
Use of services							
Hospitalized 0–24 months. No (%)	20 (44)		16 (67)		4 (19)		.001* c)
No. of hospitalizations 0–24 months. Median (mean)	0 (1.2)		1 (2.1)		0 (0.2)		.001* b)
	Min	Max	Min	Max	Min	Max	
	0	16	0	16	0	2	
Days inpatient 0–24 months. Median (mean)	0 (55.8)		25.5 (92.2)		0 (14.2)		.001* b)
	Min	Max	Min	Max	Min	Max	
	0	295	0	295	0	235	
Involuntary admitted 0–24 months. No. (%)	18 (40)		13 (54)		5 (24)		.045* c)
Days involuntary admitted 0–24 months. Median (mean)	0 (41.9)		10.5 (63.5)		0 (17.2)		.025* b)
	Min	Max	Min	Max	Min	Max	
	0	294	0	294	0	236	
Outpatient coercion 0–24 months. No. (%)	6 (13)		6 (25)		0 (0)		.015* c)
Days outpatient coercion 0–24 months. Median (mean)	0 (47.0)		0 (88.1)		0 (0.00)		.015* b)
	Min	Max	Min	Max	Min	Max	
	0	560	0	560	0	0	
Treatment							
Integrated treatment No. (%)	28 (62)		12 (50)		16 (76)		.078 c)
Good adherence with oral or depot antipsychotics. No. (%)	32 (71)		14 (58)		18 (86)		.049* c)
Good adherence with psychosocial treatment No. (%)	41 (91)		21 (87.5)		20 (95)		.47 c)

*GAF* Global assessment of function

*BPRS* Brief Psychiatric Rating Scale

a) *T*-test, b) Mann-Whitney test c) Exact unconditional z-pooled test

*Statistically significant

### Regression analysis

Female gender and high score on BPRS and low score on GAF were significant baseline predictors of becoming a frequent user in the logistic regression analysis, see Table 5, both unadjusted and adjusted for age, gender and treatment group.

**Table 5:** Logistic regression analysis of baseline and 2 year predictors for long time use of services

	OR unadjusted		OR adjusted for age, gender and treatment group	
	Estimate (CI)	*P*-value	Estimate (CI)	*P*-value
Predictors baseline				
Age (years)	0.94 (0.82 to 1.07)	0.36	0.962 (0.833 to 1.11)	0.599
Male gender	0.26 (0.073 to 0.96)	0.043^a^	0.25 (0.064 to 0.970)	0.045^a^
BPRS baseline	1.11 (1.01 to 1.21)	0.028^a^	1.10 (0.997–1.121)	0.057
Days as inpatient 1 year preceding baseline	0.998 (0.992 to 1.004)	0.44	0.998 (0.992 to 1.004)	0.496
Negative symptoms	0.889 (0.727–1.088)	0.253	0.855 (0.677–1.080)	0.19
GAF baseline	0.913 (0.854–0.982)	0.013^a^	0.92 (0.857–0.989)	0.024^a^
Predictors 2 years				
BPRS 2 years	1.10 (1.02 to 1.19)	0.013^a^	1.1 (1.006 to 1.20)	0.036^a^
Days as inpatient 2 years after baseline	1.013 (1.002–1.024)	0.023^a^	1.010 (0.999 to 1.022)	0,082
Integrated treatment or Treatment as usual	0.31 (0.087 to 1.13)	0.076	0.29 (0.073 to 1.14)	0.077
Admitted to hospital the first 2 years after baseline	8.5 (2.137–33.814)	0.002^a^	10.411 (1.956–55.399)	0.006^a^
Negative symptoms	0.935 (0.773–1.129)	0.484	0.845 (0.673–1.060)	0.146
GAF	0.950 (0.912–0.991)	0.017^a^	0.949 (0.907–0.993)	0.023^a^
Compliance medication	4.286 (0.988–18.586)	0.052	0.097 (0.015–0.607)	0.013^a^
Absence of psychotic relapse	0.165 (0.045–0.600)	0.006^a^	0.135 (0.029–0.632)	0.010^a^

*BPRS* Brief Psychiatric Rating Scale

*GAF* Global Assessment of Function

^a^significant results

After the 2 years treatment trial, score on BPRS, score on GAF, being admitted to hospital and having a psychotic relapse were all significant predictors for being a frequent user.

As an alternative, the analyses were carried out in linear regression. In linear regression, the dependent variable number of days was square root transformed to make the residuals approximately normally distributed. Results (data no shown) were similar to those in logistic regression.

In an attempt to identify patients in high risk of long-term frequent or extensive use of services we constructed a “high risk group” from categorical variables showing the most profound difference between groups during the 2 year treatment trial; suicide attempt, outpatient coercion and being hospitalised. 22 patients, 75% of frequent users and all of extensive users, fulfilled the inclusion criteria for the “high risk group”. See Table 6.

**Table 6:** Prediction of being a frequent user of inpatient days during 12 years. Divided in in a high risk group defined as being admitted or having a suicide attempt or outpatient coercion the preceding 2 years of treatment

	User group	
	Frequent user	Non frequent user
High risk group	18	4
Low risk group	6	17

Sensitivity 75%, Specificity 81%, Positive predictive value 82%, Negative predictive value 74%

## Discussion

This is one of few long-term prospective studies of recent onset psychosis and the main findings are that both at baseline and at 2 years there was a remarkable overlap in the predictive value of the psychopathological indices, i.e., this kind of stability indicate that in order to gain knowledge about long term course and hospitalization costs, it may be sufficient to assess risk factors at illness onset and initial treatment response.

During the 2 year treatment trial, poorer functioning, relapses and psychiatric hospitalizations, poor compliance with medication and suicide attempts predict higher use of inpatient services in the long-term follow-up. These also suggest that the initial treatment response is important, as is insight.

Total score on BPRS both at baseline and at 2 years were significant predictors of use of services 12 years after end of trial. Earlier research has shown predictive validity of BPRS score on length of hospital stay and its applicability in identification of patients who required non-acute extended hospital care [18, 28, 29]. Our results suggest that BPRS can be used to identify patients early in the course of schizophrenia who are likely to be frequent users of inpatient services in the future. This can be helpful in planning future interventions for this group of patients in order to improve prognosis and minimize the need of psychiatric services in the future.

Several factors the two first years that usually are considered to have a negative influence on the course of schizophrenia as reduced function (GAF), psychotic relapses, being admitted, being under coercion and reduced compliance with treatment were associated with being a frequent user in the next 12 years in bivariate analyses.

Suicide attempts during intervention period showed a significant relation to use of services in the unconditional z-pooled test indicating a significant association. In the Northern Finland Birth Cohort [30] both suicide attempts and high level of symptoms at baseline predicted poor outcome. Up to 5% of patients with schizophrenia will commit suicide [31] and the estimated rate of suicide attempts are close to 10 times that rate [32]. In our sample one patient died of complications to a suicide attempt and five patients attempted suicide. This relatively low rate of suicidality compared to other studies, may be due to the inclusion criteria excluding patients with substance abuse; a known risk factor for suicide in schizophrenia [33].

Male gender was a significant predictor in the logistic regression analysis for being a non-frequent user, both unadjusted and when adjusted for age and treatment alternatives. Male gender is a known risk factor of suicidality [34], but all of the patients in the present study who attempted or committed suicide were female. Also in the literature males have consistently been found to have a less favourable course of illness than females, [23, 35, 36], but our results appear to show the opposite tendency regarding use of services. The gender differences in suicide attempts and use of services might be due to the exclusion of patients with substance abuse as in schizophrenia this is more prevalent among males [37].

A considerable number of patients, 17 (38%), were not hospitalized during the follow up period. Two other studies with 10 years follow-up of early psychosis showed that around 40% of the patients in the OPUS study had recovery or symptom remission at 10 years follow-up [38] and 46% in the ÆSOP-study had been symptom free for 2 years [39]. This is in accordance to previous research on long term outcome in schizophrenia showing good outcome in up to 50% of patients with schizophrenia [40]. In a previous report we have described that ten patients were extensive users and had more than 500 inpatient days and were responsible for 76% of all inpatient days during the follow up period (14).

By categorizing patients into a high risk group defined by being admitted or having a suicide attempt or at outpatient coercion in the first 2 years in the treatment study, a positive predictive value of 82% for being a frequent user were found. This can be a useful tool when planning future treatment strategies.

### Limitations

A sample size of 45 limited the number of variables included in the logistic regression analysis. The selection of variables included was based on previous research on prognostic factors in schizophrenia and on what we considered of clinical significance in the actual sample. Only a large sample size would have been sufficient to test all possible variables from the bivariate analysis. Other variables chosen by other assumptions might have given different results. Dichotomizing a continuous outcome variable may have reduced the statistical power to detect a relation between the independent variables and outcome [41].

Use of inpatient services may not be an optimal measure of outcome in schizophrenia and other aspects of recovery should also be considered. Clinically unstable patients and patients with substance abuse were not included in the study and this might reduce the generalizability of the study. The long observation period, the possibility to get complete data from 90% of the participants and strict selection of appropriate statistical measures are major strengths in the study.

## Conclusion

Being admitted in the start of treatment, scores on BPRS and history of suicide attempts appear to be applicable measures in predicting the extent of future use of inpatient services in schizophrenia.

## Additional file

Additional file 1: Supplementary description of methods in the original study. The methods used in the original 2 years randomised controlled trial are described here. (DOCX 17 kb)

## Abbreviations

BPRS: Brief psychiatric rating scale
GAF: Global assessment of function
IT: Integrated treatment
SD: Standard deviation
TAU: Treatment as usual
TSR: Target symptoms rating scale
