# Supplementary material for: Predictors of long term use of psychiatric services of patients with recent-onset schizophrenia: 12 years follow-up
Source: BMC Psychiatry. 2017 Jan 14;17:18. doi: 10.1186/s12888-016-1186-x (PMC5237502; doi:10.1186/s12888-016-1186-x)
Supplement: Additional file 1: — Supplementary description of methods in the original study. The methods used in the original 2 years randomised controlled trial are described here. (DOCX 17 kb) [file 12888_2016_1186_MOESM1_ESM.docx]

# Supplementary description of data in original study

## Subjects

Recent-onset patients were selected to the study if they were diagnosed as DSM-IV schizophrenia, schizoaffective or schizophreniform disorders, between 18 and 35 years, were prescribed antipsychotic drugs, did not have any substance use disorders and were not mentally retarded. Recent-onset was defined as having psychotic symptoms for less than two years. After written informed consent, 50 patients were randomized to the out-patient treatment programme. Most of the patients were referred to the project from hospital wards (57%), other out-patient clinics (23%) and primary health care practitioners (21%). Of 168 consecutive referrals, 72 were excluded for age and diagnosis, and another 46 for other reasons (most were not recent-onset). Of the 50 patients who fulfilled the inclusion criteria, 30 were randomized to receive Integrated Treatment (Assertive community treatment: experimental group) and 20 to a treatment-as-usual condition (TAU: comparison group). The patients were included after hospital discharge and when they had recovered from the psychotic episode.

Of the included patients 81 percent had schizophrenic disorders, 62 percent were males and their mean age was 25 years. Mean GAF and BPRS score at baseline was 50 and 40, respectively. 43 percent of the patients lived in high EE environments. The two groups did not differ at baseline with respect to gender, age, total score on the Brief Psychiatric Scale (BPRS), time previously hospitalized or living in high EE families. However, the patients in the IT group had higher global functioning on the GAF at baseline (t=2.3, df=48, p<.05).

## Treatment programmes

## Integrated treatment.

The patients were treated by a multidisciplinary mental health team which provided assertive community mental health treatment with a low case-load (patient-staff ratio about 1:10). Each patient had a case manager to assist with the provision of basic social support needs, and in addition received structured psychoeducation, family communication skills and problem solving skills training, neuroleptic drug management, mobile intensive crisis management provided at home, and individual cognitive-behavioural strategies for residual symptoms and disability. This approach is described in several published manuals [for example, Falloon & Fadden, 1993). In contrast to the most comprehensive programs, our team did not provide 24 hours service and did not have a vocational specialist.

## Treatment-as-usual.

This group received standardized treatment provided by the traditional out-patient mental health care system and the community mental health care services. The patients received regular clinical case management with provision of drug management, supportive housing and day care, crises management provided by the psychiatric hospitals, rehabilitative programs focusing on promoting independent living and work activity, simple and brief psychoeducation and supportive psychotherapy. Four patients in this group received less intensive follow-up and treatment and none received the problem solving training components of structured behavioural family treatment, comprehensive psychoeducation or assertive outreach follow-up as provided to the IT group. The groups did not differ significantly with respect to antipsychotic drug treatment or drug adherence during the two-year treatment period.

## Assessments.

A battery of measures was applied at baseline, 12 and 24 months. Assessment of individualized target psychotic symptoms (1-7 score) and medication side-effects was assessed monthly. Drug compliance was registered continuously. Psychopathology was assessed bimonthly by BPRS (24 item version)(Lukoff, Nuechterlein & Ventura, 1986). The BPRS ratings were made from videotaped interviews by raters who were independent and blind to treatment conditions. In addition to the biweekly ratings, the BPRS was applied every time an increase in symptoms was suspected.

A composite clinical index to define good or poor outcome was computed, based mainly on the continuous BPRS ratings. A good outcome was rated when a patient had no major episodes of any form of psychopathology, including suicide attempts, remained free from any persist psychotic symptoms, and showed continuous optimal adherence to all aspects of the treatment programme during the entire 24-month follow-up period.

## Recurrent psychotic episode criteria.

A major psychotic episode was defined either as an exacerbation of psychotic symptoms occurring after a period of remission (i.e., having no psychotic symptoms). This was confirmed by at least a two-point increase and a score of six or seven on the Target Symptom ratings scale and a score of six or seven on one of the key psychotic symptom items on the BPRS. Finally, a major episode had to be confirmed by an independent person (researcher, family member, clinician, case manager, etc.) as a significant worsening of psychotic symptomatology. A minor episode was defined in a similar way, however, the scores on target symptoms should be in the 4-5 range and follow a period of remission.

## References

Falloon, I.R.H. & Fadden, G. (1993) Integrated mental health care. Cambridge: Cambridge University Press.

Lukoff, D., Nuechterlein K.H. & Ventura J (1986). Symptom monitoring in the rehabilitation of schizophrenic patients. Schizophrenia Bulletin, 12, 594-602
